# Splenic Hematoma With Hemoperitoneum Following a Routine Colonoscopy: A Rare Post-procedural Complication

**DOI:** 10.7759/cureus.104250

**Published:** 2026-02-25

**Authors:** Nikita Maryanov, Lachlan Driver, Cassidy T Cunningham

**Affiliations:** 1 Emergency Medicine, Brown University, Providence, USA

**Keywords:** abdominal pain, colonoscopy complications, emergency management, hemoperitoneum, perisplenic hematoma, post-procedural bleeding, splenic injury, splenic rupture, trauma imaging, upper abdominal pain

## Abstract

Though rare, a perisplenic hematoma is a potentially life-threatening complication of colonoscopies that is important to identify in patients presenting with abdominal pain following the procedure. In our case, a 62-year-old female patient presented to the emergency department (ED) with abdominal pain, tachycardia, and new anemia following a routine colonoscopy. Computed tomography (CT) showed a large perisplenic hematoma with small-to-moderate hemoperitoneum. Injury to the spleen during a colonoscopy may occur due to multiple mechanisms, including adhesions, traction of attached ligaments, and direct trauma. Management of splenic injury can include conservative management, splenic artery embolization, and splenectomy. In the case of our patient, given the absence of ongoing bleeding, she was admitted to the general surgery service and monitored with serial abdominal exams and hemoglobin checks. After her initial resuscitation with blood products, she remained hemodynamically stable and was discharged from the hospital in a stable condition.

## Introduction

Colonoscopies are performed millions of times each year in the United States. While generally well-tolerated, they carry rare but potentially serious risks. Among these, splenic injury is uncommon but clinically significant. Three mechanisms of splenic injury have been implicated: (1) adhesions between adjacent organs and the splenic capsule, increasing the risk of capsular tearing, (2) traction on the splenocolic ligament, and (3) direct trauma at the splenic flexure [1]. Splenic injuries are managed according to their severity. Minor hematomas and lacerations tend to be managed conservatively, while patients with splenic rupture may require splenectomies. Additionally, some patients may require splenic artery embolization for control of their hemorrhage. Given the high morbidity and mortality, splenic injury following a colonoscopy is an important differential diagnosis to consider while examining a patient presenting with abdominal pain [2]. This case highlights the identification and treatment of a perisplenic hematoma after a routine colonoscopy, and underscores the importance of including splenic injury in the differential for post-procedural abdominal pain.

## Case presentation

A 62-year-old female patient presented to the emergency department with severe abdominal pain and distention following a screening colonoscopy performed the previous day by a board-certified gastroenterologist. Her past medical history included bladder cancer status post radical cystectomy and pelvic exenteration, rheumatoid arthritis, hypertension, hyperlipidemia, coronary artery disease, and chronic obstructive pulmonary disease. She was taking 81 mg of aspirin daily, but was not on anticoagulation. Colonoscopy revealed patchy erosions in the terminal ileum and throughout the colon, which were sampled with cold forceps biopsies. No polyps were identified during colonoscopy, and the procedure was neither difficult nor prolonged. She developed mild abdominal pain and nausea following the colonoscopy, which progressed to severe abdominal pain, distention, night sweats, and dyspnea on exertion on the day of presentation. She also reported mild hematochezia one day before and regular bowel movements on the day of presentation. In the emergency department, she was tachycardic, with her heart rate elevated to 120-130 beats/minute (reference range: 60-100 beats/minute), and her blood pressure ranged from normotensive to hypertensive. She also denied associated chest pain or shortness of breath. She received one liter of intravenous fluids (IVF) with emergency medical services. Examination revealed a distended, diffusely tender, but soft abdomen. Dark yellow urine was noted in her urostomy bag.

Laboratory studies showed acute anemia with a hemoglobin level of 7.7 g/dL, which had decreased from 13.7 g/dL one month earlier (reference range: 11.2-14.9 g/dL) and leukocytosis with a white blood cell count of 23.7 ×10³/µL (reference range: 4.2-10.0 x 10³/µL) (Table 1). 

**Table 1:** The patient's lab values on the emergency department presentation compared to the reference ranges BUN: Blood urea nitrogen; Lab values demonstrated anemia, leukocytosis, and an elevated lactic acid troponin levels as noted in the chart.

Parameter	Patient's lab value	Reference range
Hemoglobin	7.7 g/dL	11.2-14.9 g/dL
White blood cell count	23.7 x 10³/uL	4.2-10.0 x 10³/uL
Segmented neutrophils	85.50%	
Platelets	322 x 10^9^/L	168-382 x 10^9^/L
Sodium	135 mEq/L	135-145 mEq/L
Potassium	3.9 mEq/L	3.6-5.1 mEq/L
Chloride	104 mEq/L	98-110 mEq/L
CO2	17 mEq/L	20-29 mEq/L
Anion gap	14	3-13
BUN	26 mg/dL	6-24 mg/dL
Creatinine	2.09 mg/dL	0.44-1.03 mg/dL
Whole blood lactic acid	4.1 mEq/L	0.2-1.9 mEq/L
High-sensitivity troponin	74 ng/L	3-14 ng/L

**Figure 1:**
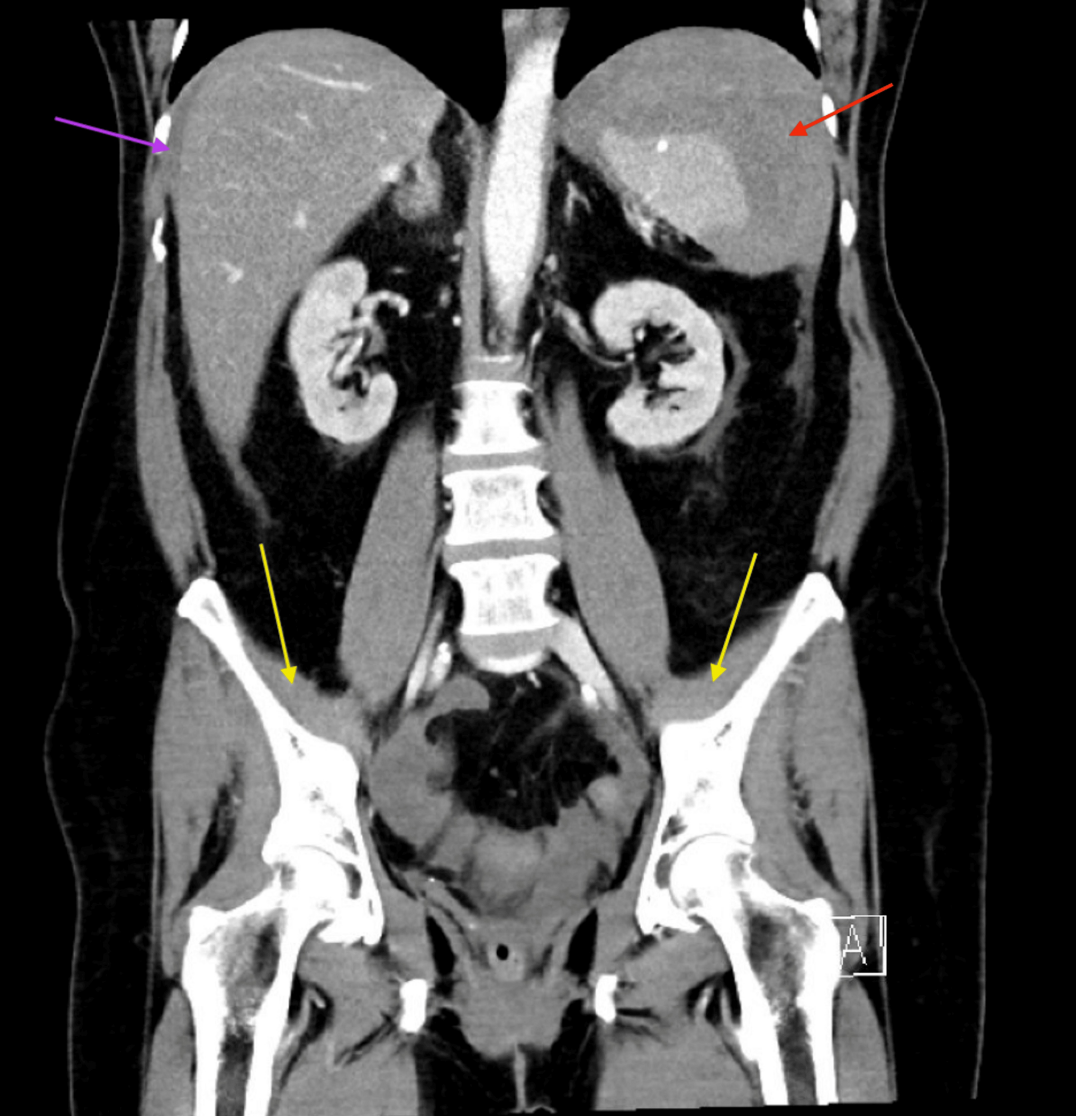
Computed tomography of the abdomen and pelvis with intravenous contrast Coronal view demonstrates a large perisplenic hematoma (red arrow) with small-to-moderate volume hemoperitoneum (yellow arrows) and blood surrounding the liver (purple arrow), without evidence of free air to suggest a bowel perforation. These findings were consistent with splenic injury, a rare but documented complication following colonoscopy.

**Figure 2:**
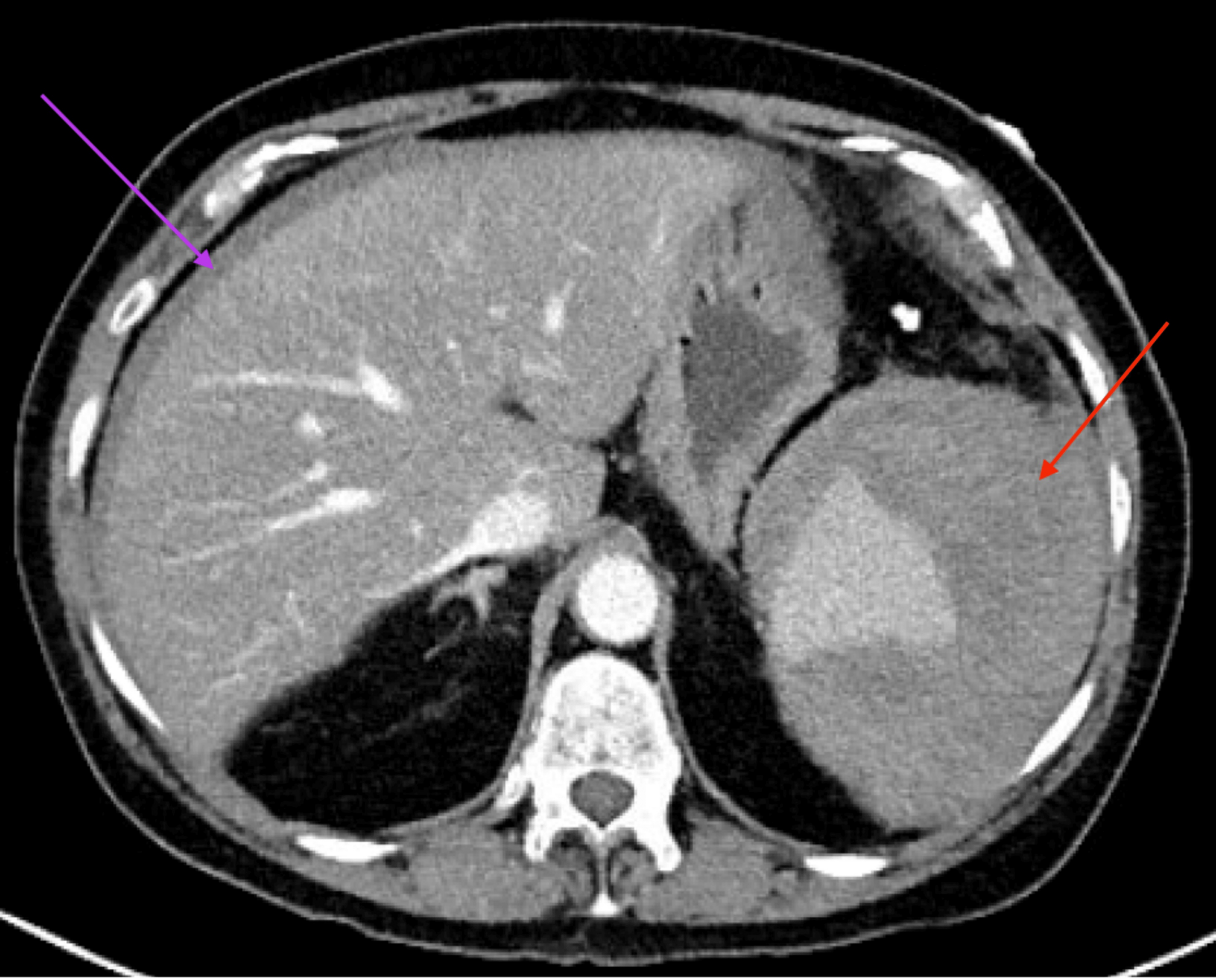
Computed tomography of the abdomen and pelvis Axial view demonstrates the perisplenic hematoma (red arrow) with associated hemoperitoneum (purple arrow).

High-sensitivity troponin was 74 ng/L (reference range: 3-14 ng/L), attributed to demand ischemia in the setting of tachycardia. The electrocardiogram (ECG) showed sinus tachycardia with no ischemic changes. Contrast-enhanced CT abdomen/pelvis demonstrated a large perisplenic hematoma (11.1 × 10.0 × 14.0 cm) with small-to-moderate hemoperitoneum and no active extravasation or pneumoperitoneum (Figures 1, 2), consistent with a grade II splenic injury, contributing to class II hemorrhagic shock.

Given the patient's hemodynamic stability after limited resuscitation (IVF and one unit of packed red blood cells), she was admitted to the surgical service for nonoperative management with close observation and serial hemoglobin checks. She did not require additional blood transfusions during her hospitalization. Over the following 24 hours, she clinically improved and maintained stable vital signs with no evidence of recurrent bleeding. She was ultimately discharged home a few days later in a stable condition. 

## Discussion

Epidemiology/risk factors

Approximately 15 million colonoscopies are performed annually in the United States [3]. Although generally safe, complications such as perforation and bleeding can occur [1,4]. Splenic injury, while rare (estimated incidence 0.001%), carries significant morbidity. Bleeding risk increases when invasive mucosal manipulation is present, with polypectomy or biopsy making up the majority of reported hemorrhagic complications. Roughly 174 cases have been reported in the literature since 1974 [5-7], with a female predominance (3:1). Injury likely occurs through traction on the splenocolic ligament, pre-existing adhesions, or direct trauma to the splenic flexure [1,4,8].

Clinical features/diagnostic workup

Most patients present within 24-48 hours of colonoscopy with abdominal pain, typically in the left upper quadrant, but often nonspecific, which can delay recognition. Kehr’s sign (left shoulder pain from diaphragmatic irritation) may be present but is neither sensitive nor specific [4,9]. Hemodynamic status varies widely, and stable vital signs do not rule out significant injury. Bedside focused assessment with sonography in trauma (FAST) ultrasound can identify free fluid, but contrast-enhanced CT remains the gold standard for diagnosis [9].

Management/outcomes

The management of splenic injuries aligns with the American Association for the Surgery of Trauma’s (AAST) Organ Injury Scale (OIS) and largely depends on the degree of splenic injury and hemodynamic status. Hemodynamically stable patients with lower-grade injuries are typically managed nonoperatively with observation, serial examinations, pain control, and transfusion as indicated [6,7,10,11]. No standardized transfusion threshold exists, so decisions should be based on clinical status and hemoglobin trends [12]. Unstable patients or those with high-grade injuries may require resuscitation with blood products and definitive intervention, such as splenic artery embolization or splenectomy. While mortality is low with prompt management, delayed recognition increases risk [6,7,10,11].

Teaching points

Splenic injury should be considered in patients, one to two days post-colonoscopy, who present with abdominal pain or discomfort, peritoneal signs, and/or hemodynamic instability [4,9]. Contrast-enhanced CT is the gold standard for diagnosing splenic injury, and a bedside FAST ultrasound may aid in diagnosis [9]. Management may include serial exams, blood product transfusion, or invasive surgical intervention, depending on the hemodynamic status and/or degree of anemia present [6,7,10,11]. Finally, a high degree of suspicion for splenic injury, combined with diagnostic imaging and appropriate resuscitation, decreases morbidity and mortality in this rare but life-threatening post-colonoscopy complication [6,7,10,11].

## Conclusions

Splenic injury is a rare but serious complication of colonoscopy, and prompt recognition is key to reducing morbidity and mortality. The risk of splenic injury increases with invasive mucosal manipulation and the performance of polypectomies and biopsies. Hemodynamic status varies widely, and stable vital signs do not rule out significant injury. Contrast-enhanced CT remains the most accurate diagnostic modality, though bedside FAST exams can detect free fluid. 

Management depends on injury severity and hemodynamic stability, from non-operative conservative management to surgical intervention, including splenic artery embolization and splenectomy. In our case, the patient underwent conservative management with serial abdominal exams and observation; her bleeding had stabilized, allowing for successful nonoperative management and discharge in a stable condition.
